# Altered metabolites in newborns with persistent pulmonary hypertension

**DOI:** 10.1038/s41390-018-0023-y

**Published:** 2018-06-12

**Authors:** Martina A. Steurer, Scott Oltman, Rebecca J. Baer, Sky Feuer, Liang Liang, Randi A. Paynter, Larry Rand, Kelli K. Ryckman, Roberta L. Keller, Laura L. Jelliffe Pawlowski

**Affiliations:** 1 Department of Pediatrics, University of California San Francisco, San Francisco, CA, USA; 2 Department of Epidemiology and Biostatistics, University of California San Francisco, San Francisco, CA, USA; 3 California Preterm Birth Initiative, University of California San Francisco, San Francisco, CA, USA; 4 Department of Pediatrics, University of California San Diego, La Jolla, CA, USA; 5 Department of Genetics, Stanford University, Palo Alto, CA, USA; 6 Department of Obstetrics, Gynecology, and Reproductive Sciences, University of California San Francisco, San Francisco, CA, USA and; 7 Department of Epidemiology, College of Public Health, University of Iowa, Iowa City, IA, USA

## Abstract

**BACKGROUND:**

There is an emerging evidence that pulmonary hypertension is associated with amino acid, carnitine, and thyroid hormone aberrations. We aimed to characterize metabolic profiles measured by the newborn screen (NBS) in infants with persistent pulmonary hypertension of the newborn (PPHN)

**METHODS:**

Nested case–control study from population-based database. Cases were infants with ICD-9 code for PPHN receiving mechanical ventilation. Controls receiving mechanical ventilation were matched 2:1 for gestational age, sex, birth weight, parenteral nutrition administration, and age at NBS collection. Infants were divided into derivation and validation datasets. A multivariable logistic regression model was derived from candidate metabolites, and the area under the receiver operator characteristic curve (AUROC) was generated from the validation dataset.

**RESULTS:**

We identified 1076 cases and 2152 controls. Four metabolites remained in the final model. Ornithine (OR 0.32, CI 0.26–0.41), tyrosine (OR 0.48, CI 0.40–0.58), and TSH 0.50 (0.45–0.55) were associated with decreased odds of PPHN; phenylalanine was associated with increased odds of PPHN (OR 4.74, CI 3.25–6.90). The AUROC was 0.772 (CI 0.737–0.807).

**CONCLUSIONS:**

In a large, population-based dataset, infants with PPHN have distinct, early metabolicprofiles. These data provide insight into the pathophysiology of PPHN, identifying potential therapeutic targets and novel biomarkers to assess the response.

## INTRODUCTION

Persistent pulmonary hypertension of the newborn (PPHN) is defined as the failure to transition from the fetal to the postnatal circulatory pattern.^1, 2^ The overall incidence is estimated at 1.8–1.9 per 1000 live births.^3, 4^ The overall mortality associated with PPHN remains relatively high at 7–15%, although mortality depends on the underlying physiology.^3, 5–7^ Clinical risk factors for PPHN include length of gestation, male sex, black race, fetal growth, maternal diabetes, obesity, and advanced age.^3, 8–11^

There is growing evidence that pulmonary vascular disease in pulmonary hypertension (PH) is associated with certain metabolic profiles. In small case–control studies, it was demonstrated that citrulline and arginine levels were lower in newborns with PPHN^12^ and in newborns with PH due to bronchopulmonary dysplasia (BPD).^13, 14^ Most recently, elevated phenylalanine and decreased tyrosine levels have been associated with PPHN.^15^ In animal studies, there is a link between carnitine metabolism and pulmonary vascular disease.^16–18^ Specifically, the disruption of carnitine homeostasis has been demonstrated in a lamb model of PH with increased pulmonary blood flow present in congenital heart disease (CHD).^16–18^ Finally, in the adult literature, an association between PH and thyroid dysfunction has been shown.^19^

In a prior study of incidence and epidemiology of PPHN, we identified the risk factors for this condition in a population-based cohort of newborns in California.^3^ The aim of the current study was to investigate metabolic dysfunction in infants with PPHN using metabolites routinely measured by the newborn screen (NBS) in a nested case–control study from our population-based cohort, to better define the biomarkers and to identify the targets for therapeutic intervention. We hypothesized that infants with PPHN would show a distinct profile of amino acid, carnitine, and thyroid stimulating hormone (TSH) levels, compared to infants without PPHN.

## METHODS

This was a nested case–control study, with the study cohort drawn from a birth cohort database maintained by the California Office of Statewide Health Planning and Development (OSHPD) containing 1,781,156 live births from the years 2007–2011, with linked maternal and infant records. This database includes detailed information on infant characteristics derived from hospital discharge records (neonatal and readmissions), linked to birth and death certificates, from birth to one year of age. Diagnosis and procedure codes are based on the International Classification of Diseases, 9th Revision, Clinical Modification (ICD-9). This database was linked to the NBS to obtain the metabolic marker data. The California Department of Public Health (CDPH) uses the NBS program to screen all newborn infants for rare inborn metabolic diseases from a heel-stick blood spot taken between 12 h and 8 days after birth. The program has been described more extensively elsewhere.^20, 21^ Infants born at ≥34 0/7 weeks’ gestational age (GA) with ICD-9 codes 747.83 (persistent fetal circulation), 416 (primary PH), or 416.8 (other secondary PH) present in birth hospitalization were considered as PPHN cases.^3^ Birth hospitalization was defined as the hospitalization from birth to initial discharge, including transfer to another hospital, if present. The same ICD-9 codes have been used to identify infants with PPHN in large administrative databases, validated by a positive predictive value (PPV) of 68.3 to 89.6%, when compared to primary medical record review.^22, 23^ To further increase the positive predictive value, an ICD-9 code for invasive mechanical ventilation needed to be present in the birth hospitalization (ICD-9 procedure codes 96.70, 96.71, 96.72, 96.7, 96.04 or diagnostic codes V46.1, V46.11, V46.12, V46.13, V46.14, 99.60, 96.05). Infants with major CHD were excluded; infants with only minor CHD associated with the diagnosis of PPHN, or diagnosed in its evaluation [e.g., ventricular septal defect, atrial septal defect, and patent ductus arteriosus] were included. We previously described the epidemiological characteristics of PPHN cases.^3^

Controls were selected randomly from those infants without PPHN who required invasive mechanical ventilation, in a 2:1 ratio. Mechanical ventilation is a surrogate marker for critical illness and as such a potentially crucial confounder. The random selection of controls guarantee a representative sample of the underlying reason for mechanical ventilation in this population-based cohort, thus removing the effect of this confounder without introducing bias. Cases and controls were further matched on confounders potentially affecting the metabolites measured by the newborn screen: gestational age, birth weight z-score, sex, parenteral nutrition (TPN) administration, and age at NBS collection.

We identified ten candidate metabolites (arginine, ornithine, citrulline, arginine to ornithine ratio, ornithine to citrulline ratio, tyrosine, phenylalanine, free carnitine, carnitine/(C16 + C18:1) ratio, and TSH) from the literature.^12–19^ Metabolites were log transformed. To avoid overfitting of the diagnostic model, the cohort of cases and controls was randomly divided into derivation (3/4 of cases with matched controls) and validation (1/4 of cases with matched controls) subsets. The derivation dataset was used to develop the diagnostic model according to the following analysis plan: each candidate metabolite was compared between cases and controls using univariable logistic regression. All metabolites with significant point estimates in the crude analysis were entered into a multivariable logistic model. Stepwise backward selection was performed by dropping the metabolites with a *p*-value > 0.05, until all metabolites were significant. This final model was tested in the validation dataset by calculating the area under the receiver operator characteristics (AUROC) curve. We performed sensitivity analyses by restricting the cases and the controls to infants not receiving TPN at the time of NBS collection.

Characteristics of cases and controls were compared by *χ^2^*, Student t-test, or Mann–Whitney test, as appropriate, with continuous data presented as mean or median with standard deviation (SD) or interquartile range (IQR). Results from logistic regression are presented as odds ratio (OR) with 95% confidence interval (CI).

All analyses were performed by using SAS version 9.4 (SAS Institute, Inc, Cary, NC) and STATA version 14.2 (Stata Statistical Software: Release 14. College Station, TX: StataCorp LP). The study was approved by the Committee for the Protection of Human Subjects within the California Health and Human Services Agency.

## RESULTS

We identified 1076 cases with an ICD-9 code, consistent with PPHN, who received invasive mechanical ventilation and had available NBS data. Overall, 2152 matched controls were randomly selected. The mean gestational age was 38 3/7 weeks in both the groups (*p* = 1.0), the mean birth weight was similar in cases and controls (3219 ± 602 g vs. 3223 ± 606 g, *p* = 0.85). Both the groups were 40.8% females (*p* = 1.0), and the proportion of infants on TPN at sample collection was 41.6% in both the groups (*p* = 1.0). Age at sample collection was 36.7 ± 16.1 h in cases and 37 ± 15.5 h in controls (*p* = 0.65, Table 1). Mothers of PPHN cases were less likely to be nulliparous, to be average weight, to have premature rupture of membranes, and to be <18-years-old. They were also more likely to have oligohydramnios and a cesarean section than mothers of controls (Table 1) Mortality was significantly higher in the PPHN cases than in the controls (9.3% vs. 4.7%, p < 0.0001).

**Table 1:** Baseline characteristics of cases and controls

N	Cases	Matched controls	p-value^*^
	1076	2152	
Matched variables			
Gestational age (weeks)			
Gestational age, mean (SD)	38.4 (1.9)	38.4 (1.9)	1.0000
<37 weeks, *n* (%)	186 (17.3)	372 (17.3)	1.0000
≥41 weeks, *n* (%)	98 (9.1)	196 (9.1)	1.0000
Birth weight			
Birth weight (g), mean (SD)	3219 (602)	3223 (606)	0.8594
SGA, *n* (%)	336 (15.6)	155 (14.4)	0.3676
LGA, *n* (%)	227 (10.6)	07 (9.9)	0.5953
Sex			
Female, *n* (%)	439 (40.8)	878 (40.8)	1.0000
Age at sample collection			
Age in hours, mean (SD)	36.7 (16.1)	37.0 (15.5)	0.6521
Parenteral nutrition at time of sample collection			
Yes, *n* (%)	448 (41.6)	896 (41.6)	1.0000
Non-matched variables			
Race			
White not Hispanic, *n* (%)	269 (25.0)	579 (26.9)	0.2463
Hispanic, *n* (%)	524 (48.7)	1,030 (47.9)	0.6539
Black, *n* (%)	66 (6.1)	100 (4.7)	0.0714
Asian, *n* (%)	149 (13.9)	260 (12.1)	0.1551
Other, *n* (%)	68 (6.3)	183 (8.5)	0.0289
Mode of delivery			
Cesarean, *n* (%)	644 (59.9)	1,016 (47.2)	<0.0001
Gestation			
Twin, *n* (%)	3 (0.3)	11 (0.5)	0.3437
Parity			
Nulliparous, *n* (%)	493 (45.8)	1,076 (50.0)	0.0250
Oligohydramnios, *n* (%)	59 (5.5)	78 (3.6)	0.0135
PROM, *n* (%)	70 (6.5)	190 (8.8)	0.0222
Chorioamnionitis, *n* (%)	65 (6.0)	165 (7.7)	0.0904
Maternal age (years)			
<18, *n* (%)	22 (2.0)	8 (3.7)	0.0104
18–34, *n* (%)	813 (75.6)	1,650 (76.7)	0.4824
>34, *n* (%)	241 (22.4)	422 (19.6)	0.0645
Maternal diabetes			
Any, *n* (%)	119 (11.1)	237 (11.0)	0.9683
Preexisting, *n* (%)	20 (2.9)	43 (2.0)	0.7872
Gestational, *n* (%)	107 (9.9)	209 (9.7)	0.8341
Maternal BMI^a^			
Underweight, *n* (%)	39 (3.6)	80 (3.7)	0.8949
Normal weight, *n* (%)	417 (38.8)	1009 (46.9)	<0.0001
Overweight, *n* (%)	266 (24.7)	502 (23.3)	0.3806
Obese, *n* (%)	229 (21.3)	396 (18.4)	0.0508
Mental illness, *n* (%)	24 (2.2)	71 (3.3)	0.0903
Smoking during pregnancy, *n* (%)	48 (4.5)	90 (4.2)	0.7120
Illicit drug use, *n* (%)	14 (1.3)	31 (1.4)	0.7501
Maternal asthma, *n* (%)	34 (3.2)	75 (3.5)	0.6296
Hypertension			
Preexisting, *n* (%)	14 (1.3)	22 (1.0)	0.4770
Gestational, *n* (%)	21 (2.0)	49 (2.3)	0.5498
Preeclampsia, *n* (%)	48 (4.5)	128 (6.0)	0.0794
Maternal asthma, *n* (%)	34 (3.2)	75 (3.5)	0.6296
Hypertension			
Preexisting, *n* (%)	31 (1.6)	48 (1.2)	0.2621
Gestational, *n* (%)	38 (2.0)	84 (2.2)	84 (2.2)
Mortality^b^, *n* (%)	100 (9.3)	101 (4.7)	<0.0001

*SD* standard deviation, *SGA* small for gestational age, *LGA* large for gestational age, *PROM* premature rupture of membranes, BMI body mass index

a Underweight: BMI <18.5 kg/m^2^; normal weight: BMI 18.5–24.9 kg/m^2^; overweight: BMI 25.0–29.9 kg/m^2^; obese: BMI ≥30.0 kg/m^2^

b According to the adequacy of prenatal care utilization (APNCU) index

^c^ Mortality: death in the first year

**Fig. 1:**
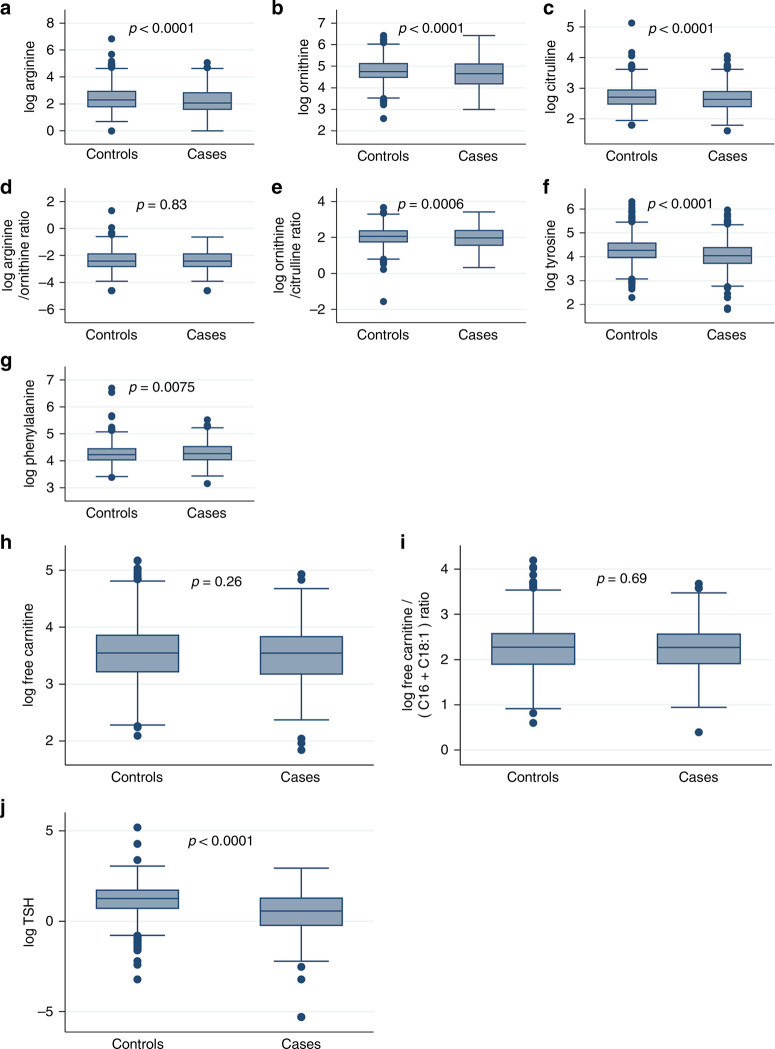
Boxplot of selected metabolites comparing cases and controls. **a** Log-transformed arginine, **b** log-transformed ornithine, **c** log- transformed citrulline, **d** log-transformed arginine/ornithine ratio, **e** log-transformed ornithine/citrulline ratio, **f** log-transformed tyrosine, **g** log-transformed phenylalanine, **h** log-transformed free carnitine, **i** log-transformed free carnitine/(acylcarnitine C16 and acylcarnitine C18) ratio, **j** log-transformed TSH

Comparison of the metabolites of interest between cases and controls in the derivation dataset show differences between the groups for amino acid and TSH levels, but no differences in free carnitine or carnitine to acylcarnitine ratio (Fig. 1).

**Fig. 2:**
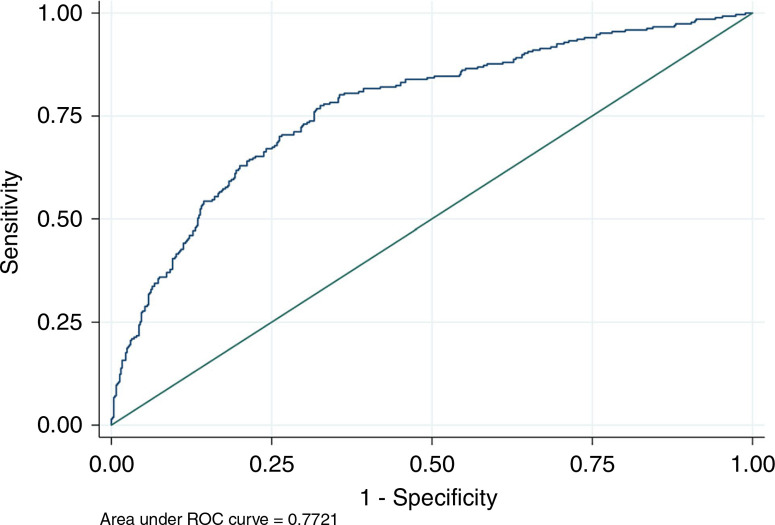
Area under the receiver operator characteristic curve (ROC) for presence or absence of PPHN

The derivation dataset consisted of 813 cases and 1626 matched controls. Comparing the cases to the controls, there was a significantly lower median for arginine, ornithine, citrulline, ornithine to cittruline ratio, and tyrosine (Table 2). The median for phenylalanine was significantly higher in the cases (Table 2). The arginine to ornithine ratio, free carnitine, and the carnitine to acylcarnitine ratio were not significantly different in the cases and the controls (Table 2). TSH was significantly lower in cases vs. controls (Table 2). After stepwise backward selection, four metabolites remained in the final model: ornithine (OR 0.32; 95% CI 0.26–0.41), tyrosine (OR 0.48; 95% CI 0.40–0.58), phenylalanine (OR 4.74; 95% CI 3.25–6.9), and TSH (OR 0.50; 95% CI 0.45–0.55). The final model was tested in the validation dataset. The AUROC curve was 0.772 (95% CI of 0.737–0.807) (Fig. 2. Using a cutoff for the predicted probability of >0.5 for PPHN classified 73.7% of newborns correctly with a sensitivity of 42.0% and a specificity of 89.5%.

**Table 2:** Univariate and multivariate associations of metabolites with PPHN in the derivation dataset

	Cases median (IQR), *n* = 813	Controls median (IQR), *n* = 1626	Crude OR (95% CI)	Adjusted OR (95% CI)
Log arginine (μmol/L)	2.08 (1.61 to 2.83)	2.30 (1.79 to 2.94)	0.81 (0.73–0.89)	—
Log ornithine (μmol/L)	4.64 (4.19 to 5.10)	4.75 (4.48 to 5.12)	0.58 (0.50–0.69)	0.32 (0.26–0.41)
Log citrulline (μmol/L)	2.64 (2.40 to 2.89)	2.71 (2.48 to 2.94)	0.56 (0.44–0.72)	—
Log tyrosine (μmol/L)	4.04 (3.74 to 4.38)	4.27 (3.97 to 4.57)	0.44 (0.37–0.52)	0.48 (0.40–0.58)
Log arginine to ornithine ratio	−2.41 (−2.81 to −1.90)	−2.41 (−2.81 to −1.90)	0.98 (0.88–1.10)	—
Log ornithine to citrulline ratio	1.97 (1.56 to 2.38)	2.06 (1.74 to 2.37)	0.71 (0.60–0.85)	—
Log phenylalanine (μmol/L)	4.27 (4.04 to 4.57)	4.23 (4.03 to 4.45)	1.40 (1.08–1.82)	4.74 (3.25–6.90)
Log-free carnitine (μmol/L)	3.54 (3.18 to 3.83)	3.55 (3.21 to 3.86)	0.88 (0.73–1.05)	—
Log carnitine/(C16 + C18:1) ratio	2.27 (1.91 to 2.56)	2.27 (1.90 to 2.57)	1.01 (0.85–1.20)	—
Log TSH (mIU/L)	0.57 (−0.22 to 1.28)	1.26 (0.72 to 1.72)	0.47 (0.43–0.52)	0.50 (0.45–0.55)

We performed a sensitivity analyses by restricting the cohort to infants who were not on TPN at the time of NBS collection (628 cases and 1256 matched controls). In contrast to the entire cohort, the univariate analyses did not show a significant difference between cases and controls for phenylalanine (OR 1.30; 95% CI 0.86–1.95), but the cases had a significant higher carnitine to acylcarnitine ratio (OR 1.36, 95%; CI 1.08–1.72). After stepwise backward selection, only three metabolites remained in the model: ornithine (OR 0.44; 95% CI 0.33–0.60), tyrosine (OR 0.47; 95% CI 0.35–0.62), and TSH (OR 0.44; 95% CI 0.38–0.51) (supplemental Table S1). The AUROC in the validation dataset was 0.783 (95% CI 0.737–0.828).

## DISCUSSION

In this population-based case–control study, we identified four metabolites routinely measured by the NBS that were associated with the presence of PPHN in our final model. Combining these four metabolites in the validation dataset was diagnostic for this condition, with an AUROC curve of 0.77. Using a cutoff of 0.5 for the predicted probability of PPHN yields a specificity of 89.5%. The selected metabolites provide insight into the potential mechanisms of this serious disorder in newborns.

The importance and potential of comprehensive metabolic profiling in PH-related conditions has recently been shown by Lewis and colleagues.^24^ They identified reproducible signatures of the right ventricular-pulmonary vascular highlighting for both new biomarkers and pathways for further functional characterization. The evidence with regards to comprehensive metabolic profiling in newborns with PPHN is less complete and should become an area of active investigation.

**Fig. 3:**
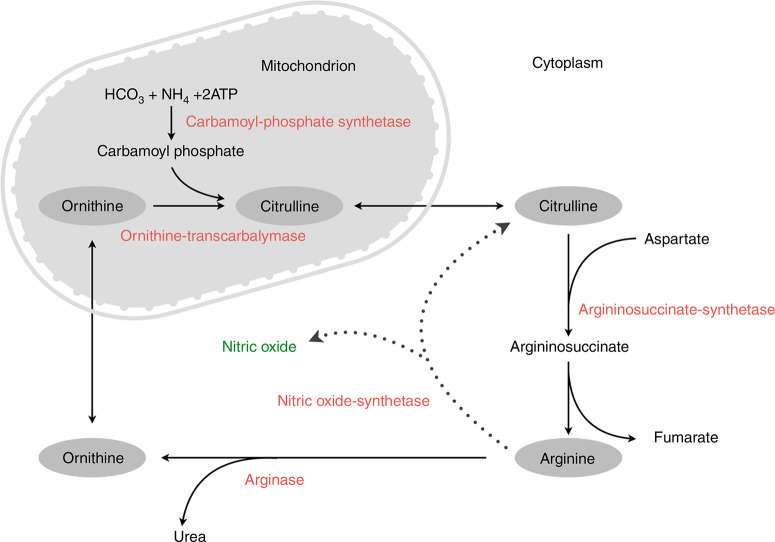
Urea cycle

Several prior studies suggest a link between PPHN and metabolism via the urea cycle;^12, 15^ endothelial cells generate the strong pulmonary vasodilator, nitric oxide (NO), from the precursor amino acid, L-arginine, supplied by the urea cycle (Fig. 3).^12^ Urea cycle enzymatic function is developmentally regulated, and at 36 weeks’ gestation, the enzymatic function is only 66–90% of adult levels.^25, 26^ Besides gestational age, genetic variations in the activity of carbamoyl-phosphate synthetase (CPS) might affect the downstream availability of the urea cycle intermediates, citrulline and arginine (Fig. 3).^12^ Pearson et al.^12^ found that newborns with PPHN had a significantly lower levels of arginine and nitric oxide metabolites, compared to infants without PPHN. Plasma citrulline levels were lower in infants with PPHN as well, although this did not reach statistical significance.^12^ In contrast, Montgomery et al.^14^ found no difference in arginine levels between very preterm infants with and without PH, while citrulline levels were significantly lower in the PH group. These differences are potentially due to different patient populations: While Montogmery et al. studied preterm infants with BPD, Pearson et al., as well as the current study included term and near term infants with PPHN. Finally, Kaluarachchi et al.^15^ found trends toward lower arginine and citrulline levels in infants with PPHN, though these did not reach statistical significance. These are all relatively small case–control studies (up to 32 cases), and none adjusted for other metabolites.^12, 14, 15^ In the urea cycle, ornithine is the product of arginine hydrolysis by arginase.^27^ To our knowledge, there are no published data on the relationship of ornithine levels to PPHN, however, there is some evidence linking decreased ornithine levels to PH among adults with sickle cell disease.^28^ In crude analysis, we found significantly lower levels of all three amino acids involved in the urea cycle in PPHN cases. After adjusting for all metabolites significant in the univariable analysis, both arginine and citrulline lost their diagnostic value and the OR for ornithine decreased to almost half, doubling the effect size in the final model. This constellation of findings is consistent with decreased CPS activity, as suggested by Pearson et al.^12^, leading to low substrate for the urea cycle and low levels of all amino acids involved in the cycle. In fact, some investigators have suggested citrulline replacement as a strategy to increase NO production in PH.^29^ However, as the end product of the urea cycle, ornithine levels have the best diagnostic value after adjusting for the other metabolites.

Kaluarachchi et al.^15^ evaluated several amino acid levels in 32 infants with PPHN and 64 controls. They found higher phenylalanine levels and lower tyrosine levels in infants with PPHN, compared to the controls. We replicated these findings in this larger study. Lower tyrosine levels in infants with PPHN might be related to oxidative stress. Experimental evidence suggests that overproduction of partially reduced reactive oxygen species— superoxide and hydrogen peroxide—contributes to the pathophysiology of PPHN,^30, 31^ and increased superoxide generation has been associated with PH in lamb models.^30, 31^ Superoxide and nitric oxide (NO) produce peroxynitrite, which reacts with tyrosine to form 3-nitrotyrosine.^32^ Peroxynitrite and 3-nitrotyrosine have been proposed as a biomarkers for oxidative distress in several diseases.^33, 34^ We speculate that high peroxynitrite levels in infants with PPHN lead to higher 3-nitrotyrosine levels and consequently, lower tyrosine concentration in this patient population. Thus, interventions focused on reducing oxidative stress and its effects may be beneficial in mitigating PPHN physiology.

Phenylalanine is converted to tyrosine by phenylalanine hydroxylase (PAH).^35^ The constellation of low tyrosine and high phenylalanine levels in infants with PPHN could be explained by impaired PAH activity. PAH activity is dependent on tetrahydrobiopterin (BH4).^35^ BH4 is a reducing substance, which may be depleted by oxidative stress.^36, 37^ We speculate that higher phenylalanine levels in infants with PPHN are a surrogate maker for depleted BH4 stores. Further, BH4 is a cofactor for endothelial NO synthase, and it has been shown to be essential for maintaining pulmonary vascular homeostasis and is a critical mediator in the pathogenesis of PH.^37, 38^ Future studies should evaluate BH4 levels in infants with PPHN and investigate its potential as a novel therapeutic target.^39^

We did not find differences in free carnitine and carnitine/ acylcarnitine (C16 + C18:1) levels between cases and controls. In contrast, in a lamb model with pulmonary vascular disease induced by increased pulmonary blood flow with a shunt, free carnitine levels were decreased and acylcarnitine levels were increased, with similar findings in children with CHD.^16, 40^ However, the pathophysiology of PPHN is that of decreased pulmonary blood flow, which may be an explanation for our contrasting findings. A recent study in adults PH found that greater concentrations of long-chain acylcarnitines were strongly associated with PH across all hemodynamic subtypes.^41^ Further investigations should focus on the acylcarnitine profiles in newborns with different etiologies of PH.

In adults, there is an association between thyroid disease and PH. Although the incidence of hypothyroidism in patients with PH is higher than the general population,^19, 42^ thyrotoxicosis can be present as a pulmonary hypertensive crisis.^42^ To our knowledge, this is the first study reporting an association between TSH and PPHN, as cases had significantly lower TSH than controls. This could be due to mild central hypothyroidism in infants with PPHN. Alternatively, higher triiodothyronine (T3) or thyroxine (T4) levels could suppress TSH in PPHN. Recently, Leeuwen et al.^43^ investigated thyroid homeostasis in newborns requiring extracorporeal membrane oxygenation (ECMO) for respiratory failure, with or without PPHN. In contrast to our findings, they found that a high incidence of abnormal thyroid screening tests, both before and during ECMO, support with normal TSH and low T4 levels. However, the results from these severely affected infants were not directly compared to those of infants without PPHN, who were also ill, and the investigators presented only dichotomized TSH levels (≤7 vs. >7mU/L).^43^ Further studies are needed to investigate thyroid homeostasis and its contribution to clinical illness in this patient population.

There are several limitations of this study. First, since we cannot determine if the infant was receiving inhaled NO (iNO) when the metabolite sample was collected, we cannot evaluate the influence of iNO administration on the metabolic profiles. In the current era, many infants with PPHN receive iNO, an accepted therapy for PH.^5, 7, 44^ However, the initiation of iNO is variable, as some hospitals where these patients are managed may not have iNO available.^45^ With regards to the classification of cases and controls, in an administrative dataset, correct ascertainment of the diagnosis is challenging. Although the ICD-9 codes we used to identify PPHN have been used by other groups and have been validated by primary chart review,^22, 23^ we cannot exclude some misclassification of the diagnosis; either some cases were missed or ICD-9 codes for PPHN have been overused, and some cases actually do not have PPHN. However, the bias induced by such non-differential misclassification would be directed toward the null, and unbiased point estimates would be expected to be even more significant. We were also unable to assess the degree of severity of PPHN in this dataset, but we did restrict our analyses to only those infants receiving mechanical ventilation, a more severe group.^3^ Another confounder that we were not able to assess in detail is TPN. While we matched the cases and the controls based on TPN administration, we did not have data on the specific component of the TPN. Most importantly, we did not have information about the administration of intralipids. Intralipid infusion has been implicated in worsening PH, possibly due to increased oxidative species. Triglycerides have also been associated with impaired endothelial function and PH in adults.^28^ We performed a sensitivity analysis by excluding infants receiving TPN at the time of NPS collection to further investigate the effect of this important confounder. Significant strengths of this study include its large, population based sample, and the novel approach of using the newborn screen to characterize PPHN. We selected metabolites based on prior studies, minimizing the risk of multiple comparisons and spurious associations. Further, we divided our data into training and validation sets to assure that our model was not over fit.

This study shows characteristic metabolic profiles in PPHN; four routinely measured substances—ornithine, tyrosine, phenylalanine, and TSH—correctly classified 73.7% of infants with respect to PPHN status. Although the timing of NBS collection cannot demonstrate which pathways and biomarkers might be responsive to effective interventions, further studies should focus on more extended analyses of the above identified pathways, and profiles relate to clinical course. This will not only improve our understanding of the pathophysiology of PPHN, but also identify potential new targets for treatment.
