# Supplementary material for: Altered metabolites in newborns with persistent pulmonary hypertension
Source: Pediatr Res. 2018 Jun 12;84:272–8. doi: 10.1038/s41390-018-0023-y (PMC7691760; doi:10.1038/s41390-018-0023-y)
Supplement: Supplementary file 1 [file CRA-84-272-s001.docx]

**Supplemental Table S1: Univariate and multivariate associations of metabolites with PPHN in derivation dataset, infants receiving parenteral nutrition at the time of NBS collection were excluded**

|  | **Cases**  **Median (IQR)**  **n = 465** | **Controls**  **Median (IQR)**  **n = 930** | **Crude OR**  **(95% CI)** | **Adjusted OR (95% CI)** |
| --- | --- | --- | --- | --- |
| **log arginine (μmol/L)** | 1.94 (1.39 to 2.30) | 2.08 (1.61 to 2.56) | 0.63 (0.54-0.75) | - |
| **log ornithine (μmol/L)** | 4.34 (3.995 to 4.72) | 4.57 (4.37 to 4.81) | 0.30 (0.26-0.39) | 0.44 (0.33-0.60) |
| **log citrulline (μmol/L)** | 2.56 (2.40 to 2.83) | 2.71 (2.48 to 2.89) | 0.43 (0.29-0.61) | - |
| **log arginine to ornithine ratio** | -2.31 (-2.81 to -1.97) | -2.41 (-2.81 to -2.04) | 1.05 (0.88-1.25) | - |
| **log ornithine to citrulline ratio** | 1.70 (1.39 to 2.04) | 1.84 (1.61 to 2.10) | 0.47 (0.35-0.62) | - |
| **log tyrosine (μmol/L)** | 4.09 (3.78 to 4.43) | 4.38 (4.14 to 4.65) | 0.27 (0.21-0.35) | 0.47 (0.35-0.62) |
| **log phenylalanine (μmol/L)** | 4.13 (3.94 to 4.33) | 4.12 (3.96 to 4.29) | 1.30 (0.86-1.95) | - |
| **log free carnitine** | 3.48 (3.17 to 3.80) | 3.49 (3.18 to 3.80) | 0.93 (0.73-1.18) | - |
| **log carnitine / (C16 + C18:1) ratio** | 2.21 (1.84 to 2.51) | 2.16 (1.75 to 2.44) | 1.36 (1.08-1.72) | - |
| **log TSH (mIU/L)** | 0.71 (-0.04 to 1.44) | 1.45 (0.93 to 1.86) | 0.38 (0.33-0.45) | 0.44 (0.38-0.51) |
